# Pharmacologic Stewardship in a Rural Community Pharmacy

**DOI:** 10.3390/healthcare11192619

**Published:** 2023-09-25

**Authors:** Chrysanthi Sardeli, Theodoros Athanasiadis, Eleni Stamoula, Dimitrios Kouvelas

**Affiliations:** Department of Clinical Pharmacology, School of Medicine, Faculty of Health Sciences, Aristotle University of Thessaloniki, GR-54124 Thessaloniki, Greece; t-athan@hotmail.com (T.A.); stamoula@auth.gr (E.S.); kouvelas@auth.gr (D.K.)

**Keywords:** compliance, medication errors, prescribing errors, drug–drug interactions, adverse drug reactions, self-medicating, community pharmacy

## Abstract

Background: Pharmacotherapy is an essential part of patient care. In order to achieve optimal health outcomes, safe and effective prescribing and administering of medications is crucial, especially since the process of pharmacotherapy can cause serious problems, mainly adverse events and/or interactions, that often pass undetected. Objective(s): To investigate the feasibility of using community pharmacies as checkpoints to detect errors and failures in prescribing, as well as patients’ compliance with pharmacotherapy. To this end, analysis and recording of the prescribing process was carried out and error-prone points were identified. Methods: Patients and caregivers filling prescriptions during the first 4 weeks of November 2017 and February 2018 answered questions in order to evaluate their attendance of regular checkups and their compliance with prescribing instructions. All prescriptions filled at the pharmacy were examined for detection of prescription errors and drug–drug interactions. Statistical analyses, including calculations of the correlation coefficient phi (φ), chi-square, and confidence intervals, were carried out. Detected errors and failures were evaluated by application of the Health Failure Mode Effect Analysis (HFMEA) quality tool. Results: A significant number of patients (16.7%) failed to regularly attend checkups regarding known health problems (95% CI: 10.6–22.7%), a corresponding percentage (16%, 95% CI: 10.1–21.9%) did not comply with prescribed pharmacotherapy, and a significant proportion of patients self-medicated regularly (32%, 95% CI: 24.5–39.5%). A total of 8.6% of prescriptions included medication combinations with a potential for severe drug–drug interactions (95% CI: 7.1–10.2%) while 58.7% of the prescriptions included combinations that could lead to moderate ones (95% CI: 56.1–61.4). The HFMEA indicated that all problems recorded required immediate interventions, except for prescribing errors. Conclusions: Community pharmacies can be potential checkpoints for the detection and evaluation of prescribing errors and pharmacotherapy failures.

## 1. Introduction

Pharmacotherapy is an essential, integral part of patient care, improving symptoms and quality of life, curing diseases, and saving lives [1]. To achieve optimal health outcomes, safe and effective prescribing and administering of medications is crucial. However, the process of pharmacotherapy can cause many serious problems. An aging population, increased drug usage, the discovery of new medicines, and the ease of self-medicating (with medicines or non-medicinal products) are some of the factors increasing the number of pharmacotherapy-related problems [2]. These problems, mainly adverse events and/or interactions, often pass undetected, especially in elderly patients [3]. This situation is rather pronounced, and a large proportion of patients, including pediatric populations, end up hospitalized due to pharmacotherapy-related problems [4]. It has been observed that 3% of hospital admissions are related to drug–drug interactions [5], while 1.33% of drugs administered to patients cause life-threatening situations [6]. In addition, poor compliance impedes the improvement of clinical outcomes and the reduction of adverse effects [1]. Studies show that significant problems include improper drug delivery, dosing errors, erroneous dosing schedules or duration of administration, etc. [7]. There are limited data regarding the occurrence of such problems in Greece; however, there is no reason to believe that Greek patients are immune and, thus, it is important to define the extent of the problem and reduce its burden.

Properly prescribing and administering pharmacotherapy and maximizing its benefits involves many stakeholders. The complexity of the process and the factors that affect its implementation allow for numerous problems and errors. The identification and analysis of such problems and errors is an important step in avoiding and correcting them, and a prerequisite for upgrading the quality of the whole process. Various process checkpoints have been employed internationally to limit negative impact. Community pharmacies might be useful in reducing errors related to pharmacotherapy because they are a daily contact point for everyone involved [8]. 

Compliance with prescribed pharmacotherapy is a significant problem of recognized importance, especially in the elderly [3]. Patient compliance is defined as “the extent to which patients follow medical advice”—that is, the extent to which the patient’s behavior, i.e., taking their medication, following a special diet, or changing their lifestyle, is in accordance with health experts’ recommendations [9]. Studies reveal a number of problems caused by poor compliance with pharmacotherapy [7]. Patients suffering from chronic diseases comply with pharmacotherapy at an estimated 50%. In cases of patients suffering from asthma, compliance is limited to 43% [9]. Compliance with cardiovascular disease preventing medications is estimated to be about 57%, regardless of the side effects that may occur [10]. 

Drug–drug interactions and the side effects related to their occurrence represent major problems that can prevent effective treatment of patients [11]. Drug–drug interactions are defined as a change in the pharmacological activity of a drug occurring due to the previous or concurrent administration of another drug, or because the action of a drug changes due to the presence of another drug in the body [12]. The total number of drugs prescribed to patients increases as they age; so, the likelihood of drug–drug interactions is higher in older people [13]. 

Errors in pharmacotherapy are defined as any avoidable action that results in inappropriate use of medicines or that causes harm to patients. Errors in pharmacotherapy can be related to any practices or procedures performed by healthcare professionals, such as prescribing, communication, packaging, distribution and delivery, education, and monitoring [14]. It has been observed that errors in pharmacotherapy can occur at any one of three main steps of the process, i.e., prescribing, dispensing, and administering. Attempts have been made to record, calculate, and evaluate such errors [11]. At least 15 types of failures have been recorded in the process. The most common mistakes observed are wrong dosage, overdosing, wrong medication, wrong drug delivery, known allergy, missed doses, wrong time of administration, wrong frequency, wrong technique, drug interactions, wrong route of administration, drug preparation errors, and inadequate monitoring [15].

Daily pharmacy practice reveals that community pharmacies come in daily contact with various pharmacotherapy-related problems, errors, and failures [16]. A community pharmacy has access to patients’ medication history and an accurate picture of any self-medicating practices that patients engage in [17]. When reviewing the problems and errors that can be detected in a community pharmacy, it becomes apparent that a significant number of those are likely to be clinically significant and are of importance regarding both patient safety and effectiveness of treatment [18].

Total Quality Management (TQM) methods can be applied to identify and prevent health-related problems. A recently published review pinpoints all aspects of health-related issues that can be handled by quality tools [19]. Studies have shown the importance of applying quality control techniques in improving pharmacotherapy and in reducing errors. Using quality systems can prevent errors at any step of the pharmacotherapy process and improve patients’ safety [20]. Detection and recording of adverse drug reactions and errors in pharmacotherapy are essential for the improvement of treatment outcomes [21]. TQM techniques can also be applied in drug usage guidelines [22].

The newly introduced Failure Mode Effect Analysis (FMEA) in healthcare procedures detects failures and evaluates their severity. Recent studies have shown that FMEA can improve patients’ pharmacotherapy [23]. Drug usage problems can be recorded and analyzed in the entirety of the process [24]. Prescribing and administration records are necessary for the implementation of FMEA and the detection of possible errors [25]. Risk assessment techniques can also be applied in the identification of drug interaction problems [26]. Other studies assess—via means of a Health Failure Mode Effect Analysis (HFMEA)—patients’ compliance with pharmacotherapy and identify possible failures [27].

This study was undertaken in order to investigate whether a community pharmacy can identify errors and failures in the prescription and administration of medicines, evaluate severity and assess whether additional checks and interventions are needed, as well as initiate appropriate corrective measures accordingly.

## 2. Methods

A rural community pharmacy was used as a checkpoint for this study. All prescriptions filled at the pharmacy during 28 consecutive days in November 2017 and February 2018 were collected for analysis, excluding those containing vaccines, and pediatric and ophthalmic preparations. The two study periods were chosen randomly. The study included questioning 150 adult patients or their caretakers about pharmacotherapy-related habits. Verbal informed consent was obtained prior to the interview.

Results were evaluated using Healthcare Failure Mode Effect Analysis (HFMEA). HFMEA is a two-stage process for failure assessment. The severity value (severity, S) and incidence rate (occurrence, O) of a failure were defined. The product of the two values, (S) × (O), known as RPN (Risk Priority Number), yielded a value to estimate criticality of failure [28]. The exact process of how the analysis was carried out is described here: initially, an accurate record of the prescribing process was created, according to the available literature [29], to identify steps where failures and errors might occur. Correspondingly, literature data were used for the application of non-numerical quality tools to the pharmacotherapy process (flowchart, fault tree analysis, and cause–effect diagram) [30], and the main points of pharmacotherapy-related failures or errors that can detected be in a community pharmacy were defined. The completion of the HFMEA study included five steps:

- Step 1: Determine the issue in question (evaluation of failures in the pharmacotherapy of patients with checkpoint at the district pharmacy).
- Step 2: Define a working group (pharmacist, pharmacy staff).
- Step 3: Create a graphic representation of the process.
- Step 4: Perform analysis of the results.
- Step 5: Draw conclusions and describe preventive actions.

During the two selected study periods, the first 150 customers of the pharmacy (adult patients or caretakers filling prescriptions) were asked by the pharmacy staff to answer questions concerning their attitudes towards prescribing and pharmacotherapy. Participation was optional. No customers asked refused participation. Questions included information about general health state, compliance with pharmacotherapy, and self-medicating. The questionnaire used is presented in Table 1.

In the first two sets of questions, correlation coefficient phi (φ) was used to correlate responses between two questions included in each category. Phi coefficient measures association for two binary variables—in this case, whether a positive answer to one question implies a positive answer in the other. The percentage of patients who responded negatively to both questions of each group was considered a measure of non-compliance. The percentage of patients who did not comply or take self-medication products and corresponding confidence intervals for each set of questions were also calculated.

The prescriptions collected were screened by the first author (a licensed pharmacist and owner of the community pharmacy) to identify possible drug–drug interactions. For this purpose, all medicines prescribed to any given patient were tested using their medication records. Interactions were ranked as severe or moderate according to their severity. Testing was performed using web-based computing tools: Medscape and Drugs.com. The number of interactions detected were compared using chi-square (x^2^) to calculate the relationship between two qualitative variables. Percentages of interactions per prescription and corresponding confidence intervals were also calculated. 

The same process was repeated in the evaluation of possible prescribing errors. All errors in dosage, dosing intervals, duration of administration, route of administration, and disease coding were identified. The numbers of errors in the two study periods were compared to ascertain whether a seasonal difference exists using the calculated chi-square (x^2^) and confidence intervals. 

Finally, using the data about detected prescribing errors and their possible impact on patient health, HFMEA tables were created, and assessment ensued to ascertain whether any additional intervention was indicated. Table 2 and Table 3 show the scales of severity and occurrence used in this project. The lower bounds of confidence intervals were used as the measure of occurrence of the incidence of interactions or errors detected in the prescriptions. The occurrence of patient non-compliance was estimated using the same method. Severity of failure was calculated using data from the literature [31].

A severity scoring scale was used to illustrate a typical ranking of events in an HFMEA study where the various categories of events are defined as follows:

- Catastrophic event—when death or permanent damage to the patient is caused;
- Serious event—when permanent physical weakness is caused, surgical intervention or days of hospitalization are increased;
- Moderate event—when level of patient care is increased;
- Mild event—when the patient is not significantly harmed.

A comparison of the data from the two study periods was conducted to investigate whether the results were a product of spurious correlations. Similarly, a correlation test between the responses to the questions asked by the pharmacy staff was conducted to examine whether the answer to a single question in each group could be a measure of patient behavior control.

All statistical analyses were performed using the SPSS software package (ΙΒΜ SPSS Statistics 25.0). Values are presented as percentages and means. The significance level was set at 5%.

## 3. Results

**Figure 1:**
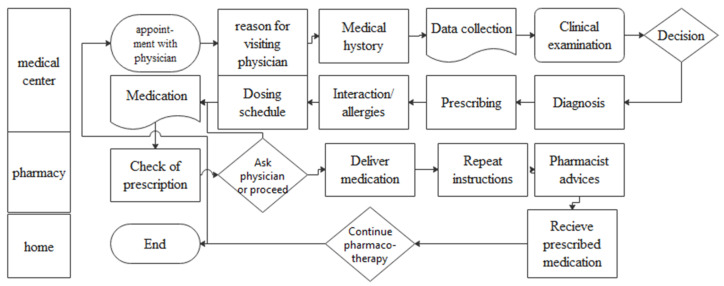
Prescribing process flowchart showing the various steps of the process and their interconnectedness.

**Figure 2:**
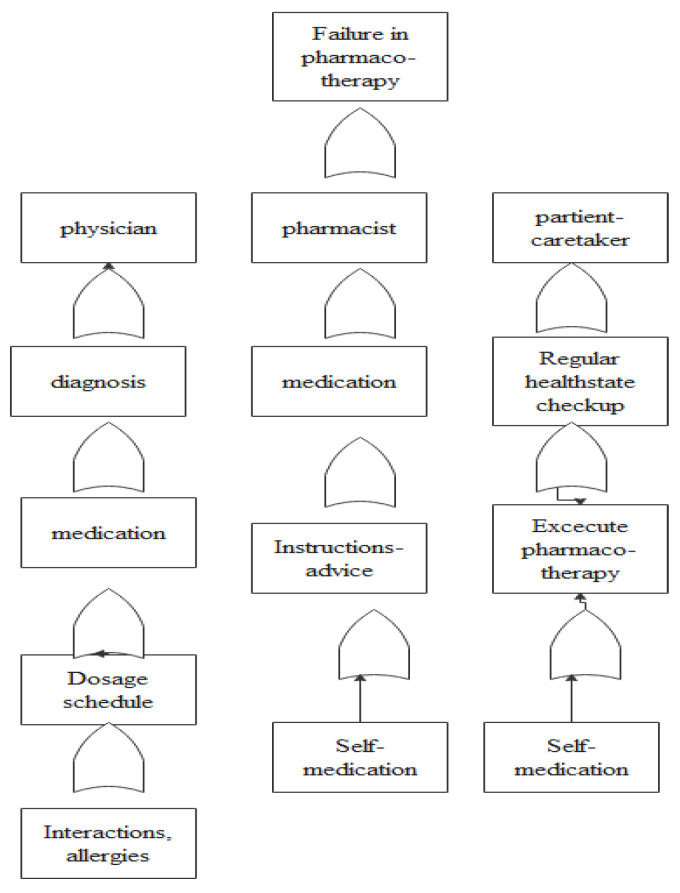
Prescribing process—fault tree analysis.

**Figure 3:**
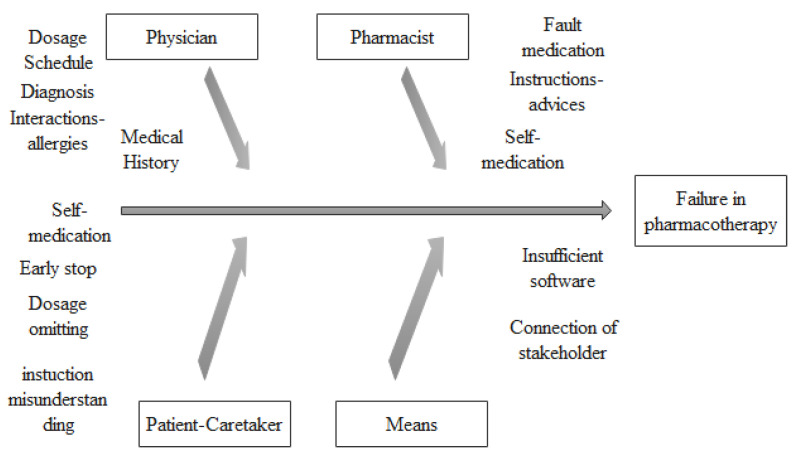
Prescribing process—cause and effect diagram.

To detect failure points, an accurate mapping of the pharmacotherapy process on a flowchart was created (shown in Figure 1). Additionally, fault tree analysis (FTA) (Figure 2) was conducted to help locate errors and a cause–effect diagram (Figure 3) was created to identify the main failures related to the process [32]. The above analysis revealed that (i) patient compliance, (ii) treatment, (iii) drug–drug interactions, and (iv) errors in prescribing are system failure points that warrant further investigation. 

### 3.1. Patients and Caretakers’ Responses

Correlating of responses to questions concerning regular checkups, using the coefficient phi (φ), indicated a moderate correlation (φ = 0.57 < 0.7). Twenty-five responders (16.7%, 95% CI 10.6–22.7%) replied that they are not regularly examined by a physician and they do not monitor their illness often. Forty-nine out of one-hundred and fifty responders did not visit their doctor every year and twenty-eight did not get their health indicators checked annually. Answers to questions evaluating compliance showed no correlation (φ = 0.19 < 0.3). The number of patients missing more than three doses a month and who did not always buy all their medications was used as a measure of non-compliance. Twenty-four out of one-hundred and fifty patients were non-compliant, which is 16% of the total responders (95% CI 10.1–21.9%). Seventy-six patients (50.6%) admitted to omitting daily medication doses, while forty of them omitted more than three doses monthly. Additionally, 63 patients (42%) did not buy all their prescribed drugs each month. Forty-eight respondents (32%, 95% CI, 24.5–39.5%) reported self-medicating daily.

### 3.2. Drug–Drug Interactions

A total of 1318 prescriptions were filled during the two study periods, containing medication combinations that could lead to 114 serious (8.7%, 95% CI 7.1–10.2%) and 774 moderate interactions (58.7%, 95% CI 56.1–61.4%).

Examination of the prescriptions collected during November 2017 (N = 653) revealed 436 drug–drug interactions, with 53 of these (8.1%) ranked as serious. A total of 452 drug–drug interactions were recorded in the prescriptions collected during February 2018 (N = 665), with 61 (9.1%) of them ranked as serious. Serious and moderate drug–drug interactions are presented in detail in Table 4, and the total number of serious and moderate drug–drug interactions for each study period are presented in Table 5.

Comparing the results of the two study periods using chi-square analysis, no difference was found in any type of interaction (*p* = 0.31). The only exception was in interactions including antibiotics, where there was a statistically significant difference between the two study periods (perhaps a seasonal difference, *p* = 0.037, <0.05). Comparing the numbers of serious interactions found in the two study periods, no statistically significant differences were found (*p* = 0.5). The same holds true for the numbers of moderate interactions found, where no statistically significant difference was observed either (*p* = 0.96). Finally, the number of interactions detected showed no difference between the two study periods (*p* = 0.64).

### 3.3. Prescription Errors

In the 1318 prescriptions collected, 145 errors were detected (10.9%, 95% CI 9.2%–12.7%). During the first study period, 70 errors were detected in 653 prescriptions. These included the following:

- Twenty-seven errors in the defined daily dose—strength of the medicine (twenty-two cases related to overdosage; five cases of underdosage);
- Six errors were due to incorrect frequency of administration;
- 32 errors were related to the duration of treatment (both longer and shorter duration);
- Five deviations from the recommended dosage regimen.

Correspondingly, during the second study period, 75 errors were found in 665 prescriptions, including the following:

- Twenty-nine errors in daily dose—strength (twenty-five overdosage; four underdosage);
- Six errors in the frequency of administration;
- 32 errors in treatment duration;
- Five deviations from the recommended dosage regimen;
- Two cases of mismatched diagnosis/indications for use.

Comparing the number of errors detected between the two study periods using chi-square analysis, no statistically significant difference was detected (*p* = 0.76).

### 3.4. Health Failure Mode Effect Analysis

Based on the results recorded above, the requisite tables for the failure study were created. Table 6 shows estimates of RPN according to severity and occurrence. Table 7 presents data regarding the performance of HFMEA in the pharmacotherapy process, indicating that all checked problems require corrective intervention (RPN equal to or >8) and additional control, except for prescription errors (RPN = 6 < 8).

## 4. Discussion

The analysis of the results reveals several points worth discussing. More than one in five of the patients interviewed were not at all concerned after an initial diagnosis about their health condition and did not visit their doctor regularly. Non-compliance was observed in a large percentage of patients questioned and, again, more than one in five patients did not follow doctors’ instructions and did not use their medications correctly. Similarly, patients did not always buy their medications from the pharmacy and reported omitting more than three doses of medication per month. Two in five patients who visited the community pharmacy omitted prescribed medications from their prescriptions and did not receive them from the pharmacy. One in three patients self-medicated and many patients used self-medicating products regularly without making their doctor aware of the fact. 

Severe interactions were detected in almost one in every ten prescriptions screened. Moderate interactions were detected in more than half the prescriptions examined. These results were consistent in the two time periods studied, hinting towards a persistent phenomenon rather than an accidental finding.

Prescribing errors were similarly detected in almost one in every ten prescriptions screened. They included dosage errors (both overdosing and underdosing were detected), errors in drug administration frequency, duration of administration, and errors relating to on-label use. These errors were persistent in both time periods examined.

The two most common types of errors observed, consistent with the available literature, were regarding the use of proton-pump inhibitors (PPIs). PPIs were found to be used for longer periods than indicated in relation to the selected diagnosis and were also administered in larger daily doses than permitted. The data collected also revealed that half of patients who were in treatment with bisphosphonates had been using these drugs for more than five years. Many of them had not visited a physician recently to get assessed regarding the need to continue receiving treatment [33].

Another interesting finding is that prescribers regularly included NSAIDs and anti-hypertensive drugs in the same prescription, two frequently interacting classes of drugs, without informing the patients or their caregivers about it. A review of the relevant literature reveals that this is a persistent issue, despite physicians frequently being made aware of the fact [34].

All these findings make it clear that a community pharmacy may be a vital checkpoint for the detection and evaluation of possible errors and failures in pharmacotherapy, a statement supported by other recent publications [35,36,37].

The main advantages of using a community pharmacy as a checkpoint are ease of collection and quick accessibility of data. The ability to access prescriptions, past medication usage records, and everyday contact with patients create a clear advantage for the early detection of errors, failures, and omissions, especially in settings where electronic health records are not available. A community pharmacy can play a central role in patient safety, since it can provide feedback to physicians concerning their patients’ on- and off-label use of medications or other products with pharmacological properties.

In this study, occurrence of events was estimated via a hands-on survey and not limited to literature reviews, increasing its reliability; although, not using a validated questionnaire needs to be mentioned as a weakness. Past medication usage records were examined to detect all drug interactions. Additionally, records were used to check the reliability of responses, as they were matched with patients’ and caretakers’ answers to validate if they indeed received all prescribed drugs from the community pharmacy every month. The study also highlighted a community pharmacy’s ability to evaluate the potential risks of pharmacotherapy. Performing an HFMEA study revealed that all examined failures were significant (categorized as severe) and that corrective and preventive actions were needed, with all detected failures requiring immediate intervention. This study shows that a community pharmacy can apply quality procedures and help improve public health. The findings of this study are in accordance with existing literature stating that a community pharmacy can serve as a checkpoint for the detection and recording of errors and failures in pharmacotherapy. The assessment of patients’ non-compliance with pharmacotherapy approached the percentages mentioned in the literature. It is worth noticing that about four in ten patients omit buying all drugs in their prescriptions.

Oral responses to questions asked by the pharmacy staff might affect the reliability of the answers. Many patients were careful in their answers and presented themselves as more responsible than they really are, a conclusion supported by the mismatch in the answers recorded. Additionally, what became evident is that many patients did not seem to understand the meaning of compliance with pharmacotherapy, given that some of them responded that they were taking their medications correctly—an answer based on their own preferences and perceptions rather than the instructions given by the physician.

The study also revealed major problems in the studied process, associated both with prescribing physicians and patients. Defining the problems can be a starting point for further understanding of the issue. Many aspects remain unexplored and need answers, such as “Are there differences between urban and rural community pharmacies in Greece?” and “What is the impact of the errors and failures detected on the actual patients’ health state?”.

Recording of consequences related to long-term pharmacotherapy is an important step in improving the process. Similarly, the recording of harm caused by pharmacotherapy can be a useful tool for physicians in their daily practice. Carrying out a large-scale study, involving multiple pharmacies in data collection from rural and urban areas, can uncover prescribing errors and interactions for wider population groups and larger geographical areas. Failure causes and factors that affect them also need to be studied. These data can be used to prevent errors and address failures. Cross-referencing patient responses with other methods of assessing compliance is still a subject of study, and further research is needed in order to elucidate the causes of the failures. Likewise, errors and failures related to the staff of the pharmacy and the procedure followed must be assessed and recorded.

The fifth step of HFMEA was omitted in this study. This step requires implementation of corrective actions and re-monitoring of the process. The authors plan to initiate a new study, with a longer-term follow-up, that will include implementation of corrective actions and recording of the results of each intervention.

This observational study aimed to highlight the ability of a community pharmacy to detect and evaluate problems in pharmacotherapy and prescribing. It was not conducted to reveal common prescribing errors and the most common interactions detected in pharmacies. The small sample of patients and the short period evaluated do not allow for such conclusions to be drawn. Generalization of results requires a corresponding procedure to be applied to a greater number of pharmacies located in both rural and urban areas and for a longer period of observation. Similarly, a major point of interest would be to record and evaluate the impact of pharmacotherapy errors and failures on patient health using objective measures. The recording of hospital admissions due to errors or non-compliance of patients, as well as their long-term health effects such as renal failure, liver problems, cardiac issues, and other effects, can highlight the importance of safe and effective pharmacotherapy. The results of such a study would be of great importance for healthcare professionals and patients alike because they can contribute to better addressing failures in prescribing and pharmacotherapy and can help improve the process involved. Limiting errors and failures can increase the effectiveness of pharmacotherapy, improve patient health and safety, reduce negative consequences of pharmacotherapy, and lower social security expenditures.

## 5. Conclusions

Given that a significant number of patients appear not at all concerned after an initial diagnosis about their health condition, do not visit their doctor regularly, do not follow doctors’ instructions, and do not use their medications correctly, community pharmacies can play a crucial role as checkpoints in the detection and evaluation of prescribing errors and pharmacotherapy failures. Ease of collection and quick accessibility of data are the main advantages of using a community pharmacy as a checkpoint. The ability to access prescriptions, past medication usage records, and everyday contact with patients create a favorable environment for the early detection of errors, failures, and omissions, especially in settings where electronic health records are not yet available.

Limiting errors and failures can increase the effectiveness of pharmacotherapy, improve patient health and safety, reduce negative consequences of pharmacotherapy, and lower social security expenditures. A community pharmacy with a well-trained staff, aware of their seminal role in the process, can play a central role in patient safety, since it can provide feedback to physicians concerning their patients’ on- and off-label use of medications or other products with pharmacological properties.

## Figures and Tables

**Table 1:** Questionnaire.

Do you routinely visit your personal physician for a yearly checkup?
Do you routinely have a yearly checkup of your health state indicators?
Do you omit daily doses of your medication? If yes, are omitted doses more than three per month?
Do you buy all the medications prescribed for you?
Do you use any self-medication products besides the medications prescribed for you by your physician?

**Table 2:** Failure severity.

Category	Points
Catastrophic	4
Severe	3
Moderate	2
Mild	1

**Table 3:** Failure occurrence.

Category	Points	Occurrence
Usual	4	1 in 10
Occasional	3	1 in 400
Unusual	2	1 in 15,000
Rare	1	1 in 15,000,000

Occurrence scoring scale used to measure failure occurrence, based on frequency of detection.

**Table 4:** Number and type of serious drug–drug interactions observed in each study period.

Drug–Drug Interactions Per Pharmacologic Category		Number/November 2017	Number/February 2018
**Antiplatelets**	**PPIs**	8	9
clopidogrel	omeprazol	3	4
clopidogrel	rabeprazol	5	5
**Calcium-channel blockers**	**Statins**	7	7
amlodipine	simvastatin	7	7
**ACE inhibitors**	**Salicylic Acid**	5	4
ramipril	Acetylsalicylic acid	4	3
lisinopril	Acetylsalicylic acid	1	1
**b-blockers**	**SSRIs-SNRIs**	4	5
nebivolol	fluoxetine	2	3
propranolol	fluoxetine	1	1
propranolol	paroxetine	1	1
**ACE inhibitors**	**NSAIDs**	4	5
enalapril	naproxen	1	2
perindopril	naproxen	2	3
quinapril	celecoxib	1	0
**Statins**	**Fibrates**	2	2
atorvastatin	fenofibrate	2	2
**Angiotensin antagonists**	**ACE inhibitors**	1	1
olmesartan	quinapril	1	1
**Other drug combinations**		20	26
duloxetine	omeprazol	1	1
citalopram	apixaban	1	1
citalopram	hydroxizin	1	1
duloxetine	sertraline	1	1
venlafaxine	amitryptiline	1	1
atenolol	rivastigmine	1	1
digoxin	metoprolol	1	1
nebivolol	propranolol	1	1
esomeprazol	cilostazol	1	1
azithromycin	acenocumarol	1	2
quetiapine	levodopa	2	2
acenocumarol	allopurinol	3	3
omeprazol	digoxin	4	4
atorvastatin	clarithromycin	1	3
simvastatin	clarithromycin	0	2
rosuvastatin	clarithromycin	0	1

**Table 5:** Total number of serious and moderate drug–drug interactions in each study period.

	November	February
b-blockers	119	116
Angiotensin’s antagonists	103	100
Diuretics	68	66
Statins	51	49
Ca-Channel inhibitors	55	53
Antidiabetics	46	46
Antidepressants	46	45
NSAIDs	4	4
ACE inhibitors	30	29
Antiplatelets	23	21
Salicylic acid	38	36
Antibiotics	6	8
PPIs	11	10

**Table 6:** Health Failure Mode Effect Analysis.

	Catastrophic	Severe	Moderate	Mild
**Usual**	16	12	8	4
**Occasional**	12	9	6	3
**Unusual**	8	6	4	2
**Rare**	4	3	2	1

Occurrence to severity scoring scale.

**Table 7:** Health Failure Mode Effect Analysis—pharmacotherapy process.

	Occurrence	Severity	RPN
Regular health state check-ups	4	2	8
Compliance with medication	8	2	8
Self-medication	4	2	8
Serious D–D interactions	3	3	9
Moderate D–D interactions	4	2	8
Prescribing errors	3	2	6

## Data Availability

Data supporting reported results can be made available upon request.

## References

[1] Corotto P.S., McCarey M.M., Adams S., Khazanie P., Whellan D.J. Heart Failure Patient Adherence Epidemiology, Cause, and Treatment. http://www.sciencedirect.com/sdfe/pdf/download/eid/1-s2.0-S1551713612000906/first-page-pdf.

[2] Leape L.L., Brennan T.A., Laird N., Lawthers A.G., Localio A.R., Barnes B.A., Hebert L., Newhouse J.P., Weiler P.C., Hiatt H. (1991). The Nature of Adverse Events in Hospitalized Patients. N. Engl. J. Med..

[3] Lee V.W., Pang K.K., Hui K.C., Kwok J.C., Leung S.L., Yu D.S.F., Lee D.T.F. (2013). Medication adherence: Is it a hidden drug-related problem in hidden elderly?. Geriatr. Gerontol. Int..

[4] Dai D., Feinstein J.A., Morrison W., Zuppa A.F., Feudtner C. (2016). Epidemiology of Polypharmacy and Potential Drug–Drug Interactions Among Pediatric Patients in ICUs of U.S. Children’s Hospitals. Pediatr. Crit. Care Med..

[5] Jankel C.A., Fitterman L.K. (1993). Epidemiology of drug-drug interactions as a cause of hospital admissions. Drug Saf..

[6] Kale A., Keohane C.A., Maviglia S., Gandhi T.K., Poon E.G. (2012). Adverse drug events caused by serious medication administration errors. BMJ Qual. Saf..

[7] Keers R.N., Williams S.D., Cooke J., Ashcroft D.M. (2013). Prevalence and Nature of Medication Administration Errors in Health Care Settings: A Systematic Review of Direct Observational Evidence. Ann. Pharmacother..

[8] Milos V., Rekman E., Bondesson Å., Eriksson T., Jakobsson U., Westerlund T., Midlöv P. (2013). Improving the Quality of Pharmacotherapy in Elderly Primary Care Patients Through Medication Reviews: A Randomised Controlled Study. Drugs Aging.

[9] WHO Defining Adherence. WHO [Internet]. 2003, 1–28. https://iris.who.int/bitstream/handle/10665/42682/9241545992.pdf.

[10] Naderi S.H., Bestwick J.P., Wald D.S. (2012). Adherence to drugs that prevent cardiovascular disease: Meta-analysis on 376,162 patients. Am. J. Med..

[11] Berdot S., Sabatier B., Gillaizeau F., Caruba T., Prognon P., Durieux P. (2012). Evaluation of drug administration errors in a teaching hospital. BMC Health Serv. Res..

[12] Kaufman D.W., Kelly J.P., Rosenberg L., Anderson T.E., Mitchell A.A. (2002). Recent patterns of medication use in the ambulatory adult population of the United States: The Slone survey. JAMA.

[13] Maher R.L., Hanlon J., Hajjar E.R., Hajjar E.R. (2014). Clinical consequences of polypharmacy in elderly. Expert. Opin. Drug Saf..

[14] About Medication Errors | NCC MERP [Internet]. http://www.nccmerp.org/about-medication-errors.

[15] Hughes R.G., Blegen M.A. (2008). Medication Administration Safety [Internet]. Patient Safety and Quality: An Evidence-Based Handbook for Nurses. Agency for Healthcare Research and Quality (US). http://www.ncbi.nlm.nih.gov/pubmed/21328757.

[16] Odukoya O.K., Stone J.A., Chui M.A. (2014). E-prescribing errors in community pharmacies: Exploring consequences and contributing factors. Int. J. Med. Inform..

[17] Eickhoff C., Hämmerlein A., Griese N., Schulz M. (2012). Nature and frequency of drug-related problems in self-medication (over-the-counter drugs) in daily community pharmacy practice in Germany. Pharmacoepidemiol. Drug Saf..

[18] Becker D.E. (2011). Adverse drug interactions. Anesth. Prog..

[19] Winters-Miner L.A., Bolding P., Hill T., Nisbet B., Goldstein M., Hilbe J.M., Walton N., Miner G., Dean D. (2014). Root Cause Analysis, Six Sigma, and Overall Quality Control and Lean Concepts. Practical Predictive Analytics and Decisioning Systems for Medicine.

[20] Mosadeghrad A.M., Woldemichael A. (2017). Application of Quality Management in Promoting Patient Safety and Preventing Medical Errors. Impact of Medical Errors and Malpractice on Health Economics, Quality, and Patients Safety.

[21] Kalra J., Kalra N., Baniak N. (2013). Medical error, disclosure and patient safety: A global view of quality care. Clin. Biochem..

[22] Sapkota B. (2017). Total Quality Management Approach to Drug and Therapeutics Committee Guidelines in a Tertiary Care Government Hospital in Nepal. Int. J. Hosp. Pharm IJHP.

[23] Lago P., Bizzarri G., Scalzotto F., Parpaiola A., Amigoni A., Putoto G., Perilongo G. (2012). Use of FMEA analysis to reduce risk of errors in prescribing and administering drugs in paediatric wards: A quality improvement report. BMJ Open.

[24] De Vries T.P.G.M. (1993). Presenting clinical pharmacology and therapeutics: A problem based approach for choosing and prescribing drugs. Br. J. Clin. Pharmac..

[25] van Tilburg C.M., Leistikow I.P., Rademaker C.M.A., Bierings M.B., van Dijk A.T.H. (2006). Health Care Failure Mode and Effect Analysis: A useful proactive risk analysis in a pediatric oncology ward. Qual. Saf. Health Care.

[26] Juárez-Cedillo T., Martinez-Hernández C., Hernández-Constantino A., Garcia-Cruz J.C., Avalos-Mejia A.M., Sánchez-Hurtado L.A., Perez V.I., Hansten P.D. (2016). Clinical Weighting of Drug-Drug Interactions in Hospitalized Elderly. Basic Clin. Pharmacol. Toxicol..

[27] Ishfaq A., Javed S., Sadeeqa S. (2017). Role of Pharmacist in Prevention of Medication Errors in Hospitals and Community Settings. World J. Pharm. Pharm. Sci..

[28] Rah J.-E., Manger R.P., Yock A.D., Kim G.-Y. (2016). A comparison of two prospective risk analysis methods: Traditional FMEA and a modified healthcare FMEA. Med. Phys..

[29] Maxwell S. (2016). Writing prescriptions: How to avoid common errors. Medicine.

[30] Aronson J.K. (2009). Medication errors: What they are, how they happen, and how to avoid them. QJM.

[31] Stamatis D.H. (2014). The ASQ Pocket Guide to Failure Mode and Effect Analysis (FMEA).

[32] López-Tarjuelo J., Bouché-Babiloni A., Santos-Serra A., Morillo-Macías V., Calvo F.A., Kubyshin Y., Ferrer-Albiach C. (2014). Failure mode and effect analysis oriented to risk-reduction interventions in intraoperative electron radiation therapy: The specific impact of patient transportation, automation, and treatment planning availability. Radiother. Oncol..

[33] Owen C., Panesar P., Marks D.J., Banks M. (2014). The dangers of proton pump inhibitor therapy. Br. J. Hosp. Med..

[34] Fournier J.-P., Sommet A., Durrieu G., Poutrain J.-C., Lapeyre-Mestre M., Montastruc J.-L., French Network of Regional Pharmacovigilance Centres (2014). Drug interactions between antihypertensive drugs and non-steroidal anti-inflammatory agents: A descriptive study using the French Pharmacovigilance database. Fundam. Clin. Pharmacol..

[35] Jambrina A.M., Santomà À., Rocher A., Rams N., Cereza G., Rius P., Gironès M., Pareja C., Franch À., Rabanal M. (2022). Detection and Prevention of Medication Errors by the Network of Sentinel Pharmacies in a Southern European Region. J. Clin. Med..

[36] Pervanas H.C., Revell N., Alotaibi A.F. (2016). Evaluation of Medication Errors in Community Pharmacy Settings. J. Pharm. Technol..

[37] De Oliveira G.S., Castro-Alves L.J., Kendall M.C., McCarthy R. (2021). Effectiveness of Pharmacist Intervention to Reduce Medication Errors and Health-Care Resources Utilization After Transitions of Care: A Meta-analysis of Randomized Controlled Trials. J. Patient Saf..

